# A *Mesorhizobium japonicum* quorum sensing circuit that involves three linked genes and an unusual acyl-homoserine lactone signal

**DOI:** 10.1128/mbio.01010-23

**Published:** 2023-05-25

**Authors:** Zehui Suo, Dale A. Cummings, Aaron W. Puri, Amy L. Schaefer, E. Peter Greenberg

**Affiliations:** 1 Integrative Microbiology Research Center, South China Agricultural University, Guangzhou, China; 2 Department of Chemistry and the Henry Eyring Center for Cell and Genomes Sciences, University of Utah, Salt Lake City, Utah, USA; 3 Department of Microbiology, University of Washington, Seattle, Washington, USA; Massachusetts General Hospital, Boston, Massachusetts, USA

**Keywords:** bacterial communication, LuxI homolog, root nodule bacteria, acyl-CoA-dependent autoinducer synthase, autoinduction

## Abstract

Members of the genus *Mesorhizobium,* which are core components of the rhizosphere and specific symbionts of legume plants, possess genes for acyl-homoserine lactone (AHL) quorum sensing (QS). Here we show *Mesorhizobium japonicum* MAFF 303099 (formerly *M. loti*) synthesizes and responds to *N*-[(2*E*, 4*E*)-2,4-dodecadienoyl] homoserine lactone (2*E,* 4*E-*C_12:2_-HSL). We show that the 2*E,* 4*E-*C_12:2_-HSL QS circuit involves one of four *luxR-luxI*-type genes found in the sequenced genome of MAFF 303099. We refer to this circuit, which appears to be conserved among *Mesorhizobium* species, as R1-I1. We show that two other *Mesorhizobium* strains also produce 2*E,* 4*E-*C_12:2_-HSL. The 2*E,* 4*E-*C_12:2_-HSL is unique among known AHLs in its arrangement of two *trans* double bonds. The R1 response to 2*E,* 4*E-*C_12:2_-HSL is extremely selective in comparison with other LuxR homologs, and the *trans* double bonds appear critical for R1 signal recognition. Most well-studied LuxI-like proteins use *S*-adenosylmethionine and an acyl-acyl carrier protein as substrates for synthesis of AHLs. Others that form a subgroup of LuxI-type proteins use acyl-coenzyme A substrates rather than acyl-acyl carrier proteins. I1 clusters with the acyl-coenzyme A-type AHL synthases. We show that a gene linked to the *I1* AHL synthase is involved in the production of the QS signal. The discovery of the unique I1 product enforces the view that further study of acyl-coenzyme A-dependent LuxI homologs will expand our knowledge of AHL diversity. The involvement of an additional enzyme in AHL generation leads us to consider this system a three-component QS circuit.

**IMPORTANCE:**

We report a *Mesorhizobium japonicum* quorum sensing (QS) system involving a novel acyl-homoserine lactone (AHL) signal. This system is known to be involved in root nodule symbiosis with host plants. The chemistry of the newly described QS signal indicated that there may be a dedicated cellular enzyme involved in its synthesis in addition to the types known for production of other AHLs. Indeed, we report that an additional gene is required for synthesis of the unique signal, and we propose that this is a three-component QS circuit as opposed to the canonical two-component AHL QS circuits. The signaling system is exquisitely selective. The selectivity may be important when this species resides in the complex microbial communities around host plants and may make this system useful in various synthetic biology applications of QS circuits.

## INTRODUCTION

Many *Proteobacteria* and *Nitrospirae* species use acyl-homoserine lactones (AHLs) as quorum sensing (QS) signals. The AHLs are produced by a LuxI-type synthase and detected by a cognate LuxR-type receptor, which binds to specific DNA sequences, thus affecting gene transcription (1 - 5). In many bacteria, AHL QS is important for success in mutualist or pathogenic interactions with eukaryotic hosts (1 - 5). The list of such bacteria includes nitrogen-fixing symbionts of legume plants, including species of *Rhizobium, Bradyrhizobium, Sinorhizobium,* and *Mesorhizobium* (6 - 12). There are a few reports on QS in the genus *Mesorhizobium* (9, 13, 14), including two, which provide evidence that QS is involved in nodulation of host legume roots (11, 15).

We sought to learn about AHL QS in *Mesorhizobium japonicum* (formerly *Mesorhizobium loti* [16]) by studying a strain called MAFF 303099, which is a symbiont of the model legume *Lotus japonicu*s. This symbiotic relationship has been used to dissect plant–microbe interactions (17, 18), and several useful resources exist including the sequenced genomes of both host and symbiont (19, 20) and mutant collections (21, 22). The *M. japonicum* MAFF 303099 genome possesses four *luxI* and *luxR* homologs. We are unaware of any studies of QS in this strain, but there has been work on QS in other strains and species of *Mesorhizobium*. Homologs of the MAFF 30399 *luxI* and *luxR* genes have been given different names (9, 11, 23). As a matter of convenience, we refer to the MAFF 303099 QS genes simply as *I1-I4* and *R1-R4*. Table S1 provides information on the different *luxI* and *luxR* homologs from *Mesorhizobium* species studied to date and their homologies to the MAFF 303099 QS genes.

Information on AHLs produced and detected by different *Mesorhizobium* species and strains is fragmented. AHL synthase activities were sometimes studied by using recombinant *Escherichia coli* and sometimes by using analytical methods, which can misidentify minor AHLs as the primary AHL signal or not detect abundant AHLs. This can be problematic in the case of some LuxI homologs, which use acyl-coenzyme As (acyl-CoAs) as a substrate for AHL synthesis. Over the last several years, we have learned that there are two subgroups of LuxI homologs, those that use *S*-adenosylmethionine and fatty acyl-acyl carrier proteins (ACPs) from fatty acid biosynthesis as substrates and those that use *S-*adenosylmethionine and acyl-CoAs from a variety of different cellular metabolic pathways as substrates. Members of the CoA-utilizing subgroup can be sorted from the acyl-ACP subgroup bioinformatically (24). Because the generation of CoA substrates often requires specific acyl-CoA ligases, results with recombinant *E. coli* can lead to incorrect identification of the natural signal (24, 25). The CoA-dependent AHL synthases are often found in the *Alphaproteobacteria* (24). The first one to be described is produced by a purple photosynthetic bacterium *Rhodopseudomonas palustris*, which requires exogenous *p*-coumarate and a *p*-coumaroyl-CoA ligase to produce its QS signal *p*-coumaroyl-HSL (25). Other examples include *Prosthecomicrobium hirschii*, which uses a CoA-dependent AHL synthase to produce phenylacetyl-HSL (24). *Bradyrhizobium japonicum* uses a CoA-type AHL synthase to produce isovaleryl-HSL (7), and *Bradyrhizobium* strain ORS278 uses its CoA-type AHL synthase to produce cinnamoyl-HSL (26). Two of the *M. japonicum* I homologs (I1 and I4) sort as CoA-dependent types. It would not be surprising to find these AHL synthases produce yet undescribed AHLs in their native background.

Here we describe our use of a radiotracer analysis of AHLs produced by *M. japonicum* MAFF 303099. The radiotracer technique provides information about the relative abundances of AHLs produced under the conditions of the experiment (27). There is one major AHL produced, and it has unique features. We determine which of the four I proteins is responsible for production of this AHL, and we show that the cognate R protein responds selectively to the AHL we identified. We discovered that a gene immediately downstream of, and presumably co-transcribed with, *I1* was involved in the synthesis of the unusual AHL. Mining of genome sequence databases indicates that this R-I pair (*R1-I1*) is conserved in most sequenced *Mesorhizobium* genomes. This finding suggests that it may be particularly critical to the success of this bacterium in the rhizosphere or in the process of root nodulation.

## RESULTS

### Production of one major AHL by *M. japonicum* MAFF 303099

**Fig 1:**
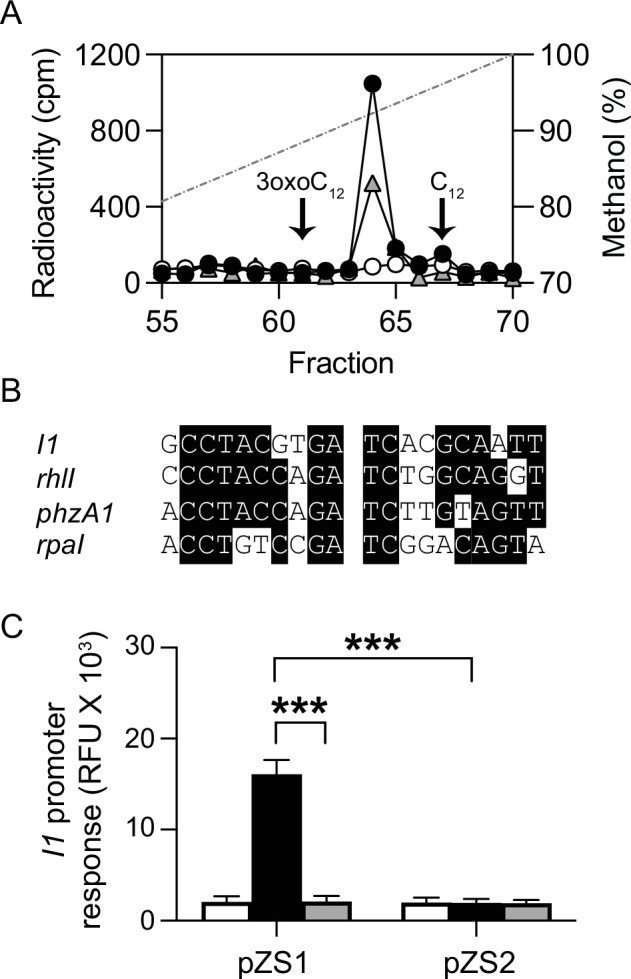
*M. japonicum* produces an AHL, which activates *I1* gene expression. (**A**) HPLC profile of ^14^C-AHLs in ethyl acetate extracts of *M. japonicum* MAFF 303099 culture fluid incubated in the presence (white circles) or absence (black circles) of AiiA lactonase or *Mesorhizobium* AP09 culture fluid (gray triangles). The fractions where chemically synthesized 3-oxo-C_12_-HSL and C_12_-HSL elute are indicated. The dashed line shows the methanol gradient. ^14^C-labeling was performed two to four times for each strain with similar results, a representative experiment is shown. (**B**) The sequence of an inverted repeat centered 61.5 bp upstream of the start codon for the *I1* open reading frame. The sequence is aligned with promoter sequences from *Pseudomonas aeruginosa* PAO1 (*rhlI* and *phzA1*) and *R. palustris* CGA009 (*rpaI*) that are known LuxR homolog binding sites (33 - 35). (**C**) Expression of *I1-mCherry* reporters (plotted as relative mCherry fluorescence units [RFU]) with no added culture fluid extracts (white bars), extracts from MAFF 303099 wild type (black bars), or extracts from an *I1* transposon mutant (gray bars). The pZS1 plasmid harbors *R1* in addition to the *I1-mCherry* fusion, and pZS2 harbors the fusion only. Data are the mean relative RFU from three biological replicates (each with duplicate technical replicates), and the error bars represent standard deviations. Significant differences (Student’s *t*-test) between samples are indicated (*P* < 0.001, ***).

Two common methods to identify AHLs are relaxed-specificity bioassays and mass spectrometry (MS). Bioassays can miss novel AHLs (7), and they can greatly overestimate the relative abundance of minor AHLs, leading to signal misidentification (27). Mass spectrometric approaches for AHL discovery generally rely on the identification of a diagnostic MS2 ion with a mass of 102 (11, 28, 29), corresponding to the conserved homoserine lactone ring. Mass spectra of most, but not all, AHLs have this MS2 fragment. Novel AHLs that do not exhibit this fragment can be missed by MS. Therefore, we used a ^14^C-AHL radiotracer assay (27, 30) that detects all AHLs synthesized by cells incubated in the presence of ^14^C-methonine regardless of the AHL structure, and it provides information about the relative abundances of the AHLs (7, 24). Logarithmic-phase cells were suspended in a buffer with glucose as an energy source and L-[1-^14^C]-methionine. The ^14^C in this position will be incorporated into the homoserine lactone ring of AHLs synthesized by a LuxI homolog. After incubation with L-[1-^14^C]-methionine, cells were removed by centrifugation, and hydrophobic compounds were extracted from the culture fluid with ethyl acetate. Extracts were fractioned by C_18_-reverse phase high-pressure liquid chromatography (HPLC) (Fig. 1A). Most of the radiolabel was eluted in a single fraction (Fig. 1A). To obtain additional evidence that the radiolabeled material was an AHL, we treated an extract with AiiA lactonase (Fig. 1A), which cleaves the homoserine lactone ring of AHL compounds (31, 32). The lactonase treatment essentially eliminated the radioactive peak (Fig. 1A).

Often this type of analysis can lead to a presumptive identification of the radioactive AHL based on HPLC retention time. We were unable to make such an assignment. The two standards that elute most closely to the peak at fraction 64 are 3-oxo-dodecanoyl homoserine lactone (3-oxo-C_12_-HSL) and dodecanoyl homoserine lactone (C_12_-HSL), which elute earlier or later than the radiolabeled material (Fig. 1A). A previous report indicated that C_12_-HSL was the product of a gene homologous to *I1* in *M. loti* NZP 2213 and that one of the other LuxI homologs (I3) directed NZP 2213 to produce shorter chain AHLs (Table S1) (11). We found a very small radioactive peak at the position where C_12_-HSL elutes in our HPLC analysis (Fig. 1A). It might be that the long-chain AHL detected previously was misidentified as C_12_-HSL or that C_12_-HSL is a minor product and a more abundant AHL was overlooked or that although the amino acid sequence of the AHL synthase from the other strain is 95% identical to I1, its reaction product is different than that of I1. We address this issue in the section below.

We focused our attention on the MAFF 303099 *I1* gene because it showed homology with the NZP 2213 *luxI* gene reported to synthesize C_12_-HSL, and the NZP 2213 *I1* is required for normal *Lotus* root nodulation (11). The genome of another *Mesorhizobium* sp., strain AP09 (36), possesses a single *luxR-luxI* gene pair, which is homologous to *R1-I1* (the *I* gene product shows 99% identity with the MAFF 303099 I1). We performed a radiotracer experiment with this strain and found that the radioactivity was eluted in the same position as the material found in the analysis of MAFF 303099 (Fig. 1A). This is an indication that the radioactive peak is the product of I1, which is a member of the acyl-CoA subgroup of LuxI homologs (see Materials and Methods).

### The *M. japonicum* MAFF 303099 I1 together with the R1 positively autoregulates the transcription of *I1*

In the great majority of LuxR-LuxI-like AHL regulatory circuits, one of the regulated genes is the *I* gene itself, resulting in AHL synthesis being positively autoregulated (37). In fact, Yang et al. (11) provided evidence to support the idea that the NZP 2213 *I1* homolog is positively autoregulated, and we identified a potential R1 DNA-binding sequence upstream of the MAFF 303099 *I1* gene (Fig. 1B). We constructed a transcription reporter plasmid (pZS1) containing both *R1* and the *I1* promoter fused to a promoterless *mCherry* and introduced this plasmid into *P. putida*, a heterologous host without its own AHL QS circuit. We have used *P. putida* to study QS circuits from other bacteria with high GC content genomes (24). When *P. putida* (pZS1) was grown in the presence of MAFF 303099 culture fluid extracts, mCherry fluorescence was about eight times that of *P. putida* grown in the absence of MAFF 303099 culture fluid extracts. Furthermore, culture fluid extracts from a MAFF 303099 *I1* transposon insertion mutant did not stimulate *mCherry* expression (Fig. 1C). This provides further evidence that I1 is responsible for the synthesis of the AHL produced by cultures of *M. japonicum*.

To test our hypothesis that the R1 protein was required for a response to the *M. japonicum* AHL, we constructed pZS2 by deletion of 578 bp of the 732 bp *R1* open reading frame. Addition of MAFF 303099 culture fluid extracts to *P. putida* (pZS2) did not affect *mCherry* expression (Fig. 1C). We conclude that I1 is responsible for the production of the AHL, and R1 is a transcription factor that is responsive to the I1-produced AHL. With the development of an assay for the undefined AHL (Fig. 1C), we then moved forward to purify and elucidate the AHL structure.

### Purification and structural determination of the *M. japonicum* MAFF 303099-produced AHL

**Fig 2:**
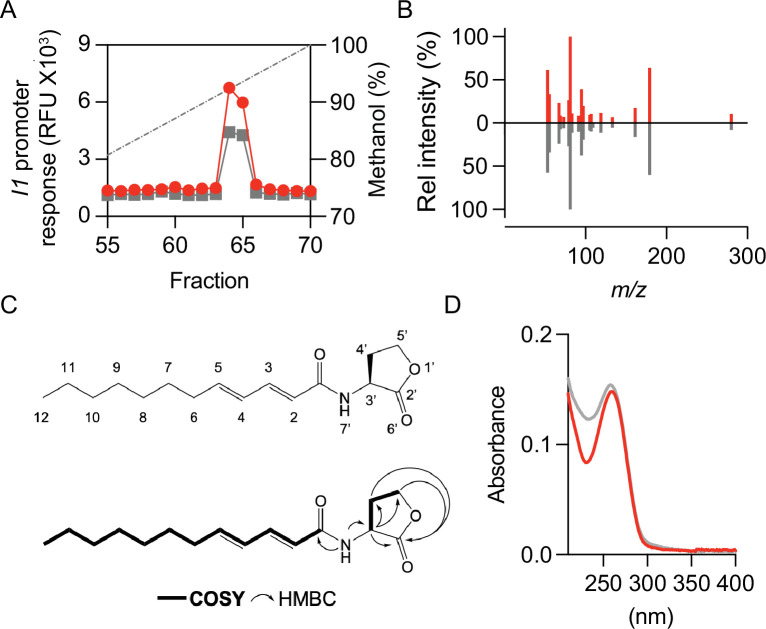
Identification of the *M. japonicum* I1 product. (**A**) HPLC analysis of culture fluid extracts from *M. japonicum* MAFF 303099 (red circles) and chemically synthesized 2*E,* 4*E*-C_12:2_-HSL (gray squares). The dashed line shows the methanol gradient. Transcription from the *I1* promoter was measured by using the *P. putida* (pZS1) bioassay. (**B**) MS/MS daughter scans of the m/z 280 (*M* + H) ion from the purified natural product (red lines) and chemically synthesized 2*E,* 4*E*-C_12:2_-HSL (gray lines). Daughter scan fragment masses and intensities are reported in Table S2. (**C**) Deduced structure of the *M. japonicum* I1-produced QS signal (top) assembled from NMR data (bottom) (Table S3; Fig. S1 through S5). Correlation spectroscopy (COSY) and heteronuclear multiple bond correlation spectroscopy (HMBC). (**D**) UV absorbance spectra of purified natural product (red) and chemically synthesized 2*E,* 4*E*-C_12:2_-HSL (gray) in methanol. RFU, relative mCherry fluorescence unit.

We used the *P. putida* (pZS1) response to MAFF 303099 culture fluid extracts for activity-guided purification of the AHL. We first showed that the HPLC profile of the bioactive material was similar to that of the radioactive material, and indeed it was eluted in fractions 64 and 65 (Fig. 2A), whereas the radioactive material was eluted in fraction 64 (Fig. 1A). We then extracted the AHL from 5 L of MAFF 303099 culture fluid with ethyl acetate and subjected the extract to gradient HPLC. Each HPLC fraction was tested for bioactivity by using *P. putida* (pZS1), and a single peak was eluted in fractions 64 and 65. Fractions 64 and 65 were pooled and subjected to an isocratic HPLC separation as described in the Materials and Methods. The single region of bioactive material was analyzed by liquid chromatography tandem MS (LC-MS/MS) and showed a product with an (*M* + H) of 280.1918. This finding indicates that the bioactive material has a molecular formula of C_16_H_25_NO_3_ (mass tolerance of 1.9 ppm), which is consistent with a di-unsaturated AHL molecule with a 12-carbon side chain. AHL compounds usually exhibit characteristic MS2 fragments (102.0550, 84.044, 74.06, and 56.06) derived from the homoserine lactone ring (28, 29). Interestingly, such MS2 fragments were not evident in our spectra (Fig. 2B; Table S2).

To determine the location and stereochemistry of the predicted double bonds in the purified AHL, we performed ^1^H, ^13^C, and various two-dimensional nuclear magnetic resonance (NMR) analyses (Table S3; Fig. S1 through S5). To improve the resolution for ^13^C-NMR analysis, we extracted and purified the AHL from an additional 2 L of the supernatant fluid from cultures grown in a minimal medium with uniformly labeled ^13^C-glucose as the sole carbon source (see Materials and Methods). NMR analyses revealed a conjugated system beginning with the carbonyl at the C1 acyl position and *trans* olefins at the C2 (*J* in Hz 15) and C4 (*J* in Hz 15.5) positions, resulting in our determination that the purified compound was *N*-[(2*E*, 4*E*)-2,4-dodecadienoyl] homoserine lactone (2*E,* 4*E*-C_12:2_-HSL, Fig. 2C). This molecule was chemically synthesized previously (38) and provided to us for comparison with our purified AHL by Stefan Schultz. The chemically synthesized 2*E,* 4*E*-C_12:2_-HSL was active in the *P. putida* (pZS1) bioassay and had an HPLC elution profile similar to the natural product (Fig. 2A). Furthermore, the LC-MS/MS profiles of the purified and synthetic AHLs were indistinguishable (Fig. 2B; Table S2). Because 2*E*, 4*E*-C_12:2_-HSL has two conjugated double bonds it should have some UV light absorbance and in fact the chemically synthesized and natural products had similar absorption spectra (two peaks with maximum absorbances at about 210 and 259 nm, Fig. 2D). We conclude that *M. japonicum* I1 catalyzes the synthesis of 2*E,* 4*E*-C_12:2_-HSL.

### *M. japonicum* MAFF 303099 produces 2*E,* 4*E*-C_12:2_-HSL in late logarithmic growth phase

**Fig 3:**
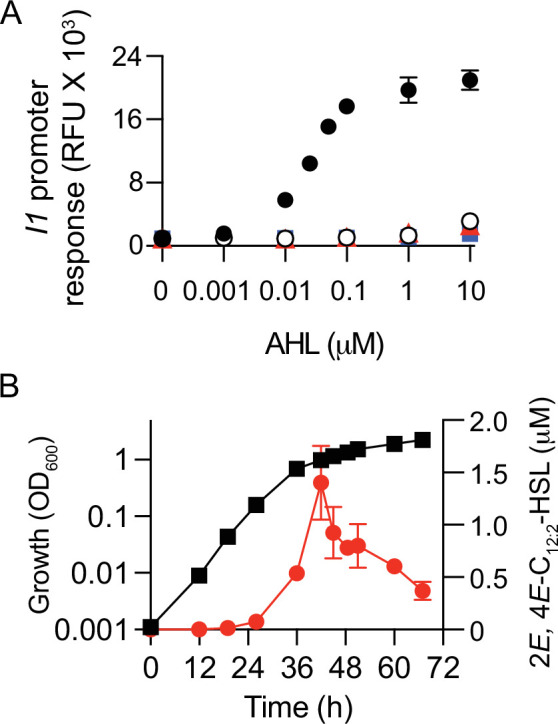
The QS signal 2*E,* 4*E*-C_12:2_-HSL is produced in late logarithmic growth, and the *trans* double bonds are essential for signaling. (**A**) Dose–response curves showing activation (or lack thereof) of the R1 receptor in *P. putida* (pZS1) by various AHL compounds: 2*E,* 4*E*-C_12:2_-HSL (black circles), 9*Z*-C_14:1_-HSL (white circles), C_12_-HSL (red triangles), and C_14_-HSL (blue squares). (**B**) 2*E,* 4*E*-C_12:2_-HSL production (red circles) during growth of *M. japonicum* MAFF 303099 in TY broth (black squares). Culture growth was measured as optical density at 600 nm (OD_600_). Data in 3A are the means and standard deviation of three to five biological replicates, and data in 3B are the means and ranges for two biological replicates, all with duplicate technical replicates.

To make quantitative determinations of the amount of 2*E*, 4*E*-C_12:2_-HSL produced by MAFF 303099, we used the chemically synthesized 2*E,* 4*E*-C_12:2_-HSL to create a standard curve based on activity in the *P. putida* (pZS1) assay (Fig. 3A). The maximum amount of 2*E,* 4*E*-C_12:2_-HSL present in culture fluid was about 1.5 µM, a concentration typical for AHL-producing bacteria. Levels of 2*E,* 4*E*-C_12:2_-HSL were low in early- and mid-logarithmic growth phase and then increased rapidly during late logarithmic growth and the transition to the stationary phase (Fig. 3B). This profile is characteristic of positively autoregulated AHL QS systems (39, 40). Note 2*E,* 4*E*-C_12:2_-HSL levels decreased later in the stationary phase (Fig. 3B). AHLs are unstable at alkaline pH (41); however, the pH of our MAFF 303099 cultures remained at 7. An alternative explanation for the AHL decrease is that MAFF 303099 produces a specific AHL-degrading enzyme(s) in the stationary phase as has been reported for other bacteria (42).

### The *M. japonicum* MAFF 303099 R1 is a particularly selective 2*E*, 4*E*-C_12:2_-HSL receptor

We used the *P. putida* (pZS1) reporter to test a panel of AHLs for an ability to activate expression from the *I1* promoter (Fig. 3A). There was a dose-dependent response to the I1 product, 2*E*, 4*E*-C_12:2_-HSL. The half-maximal response was about 25 nM, and the response was saturated at about 100 nM 2*E*, 4*E*-C_12:2_-HSL. Most LuxR homologs studied to date can respond to a variety of non-cognate AHLs, with varying degrees of selectivity (43). We screened several AHLs ranging in acyl side chain length from 10 to 14 (C_10_-HSL, C_12_-HSL, 3-oxo-C_12_-HSL, 3-hydroxy-C_12_-HSL, C_14_-HSL, 3-hydroxy-7*Z*-C_14:1_-HSL, 3-oxo-7*Z*-C_14:1_-HSL, and 9*Z-*C_14:1_-HSL) Most of these showed no activity. Two, C_12_-HSL and 9*Z-*C_14:1_-HSL, showed barely detectable activity at 10 µM, the highest concentration tested (Fig. 3A).

### *M. loti* NZP 2213 produces 2*E,* 4*E*-C_12:2_-HSL

**Fig 4:**
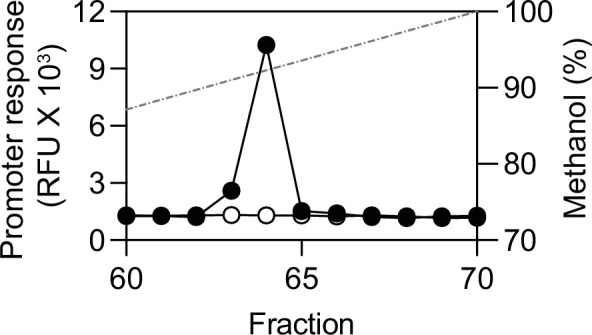
*M. loti* NZP 2213 produces 2*E,* 4*E*-C_12:2_-HSL. HPLC separation of ethyl acetate extract from 10 mL of early stationary phase NZP 2213 culture fluid. Fractions were analyzed by using the R1-specific reporter *P. putida* (pZS1) (black circles) and the QscR-specific reporter *E. coli* (pJNQ, pPROBE-P_PA1897_) (white circles). The relative fluorescence units (RFU) of mCherry (R1-specific reporter) or green fluorescence protein (QscR-specific reporter) for each fraction are plotted. The dashed line shows the methanol gradient. Three cultures were assessed with similar results, a representative experiment is shown.

As discussed above, Yang et al. showed the NZP 2213 I1 homolog was important for root nodulation, and they presented evidence to suggest the AHL product of I1 was C_12_-HSL (11). The C_12_-HSL assignment was based on a relaxed-specificity bioassay-coupled thin layer chromatography procedure and MS analysis of compounds with a MS2 fragment of 102 (11). The apparent differences between our findings and those of Yang et al. (11) might be that we have used different *Mesorhizobium* species; however, the analytical techniques employed previously (11) would have been blind to 2*E,* 4*E*-C_12:2_-HSL. We extracted ethyl acetate AHLs from culture fluids of *M. loti* NZP 2213 as the cells entered the stationary phase and used HPLC to fractionate the extracts. By using our 2*E*, 4*E*-C_12:2_-HSL-specific bioassay, we found activity, which was eluted in the position of 2*E*, 4*E*-C_12:2_-HSL, and we did not detect any activity with a C_12_-HSL bioassay (Fig. 4). We conclude that the most abundant AHL in the NZP 2213 extracts was 2*E*, 4*E*-C_12:2_-HSL. If C_12_-HSL was produced it was below the limit of detection.

### The gene adjacent to *I1* is involved in 2*E,* 4*E*-C_12:2_-HSL production

**Fig 5:**
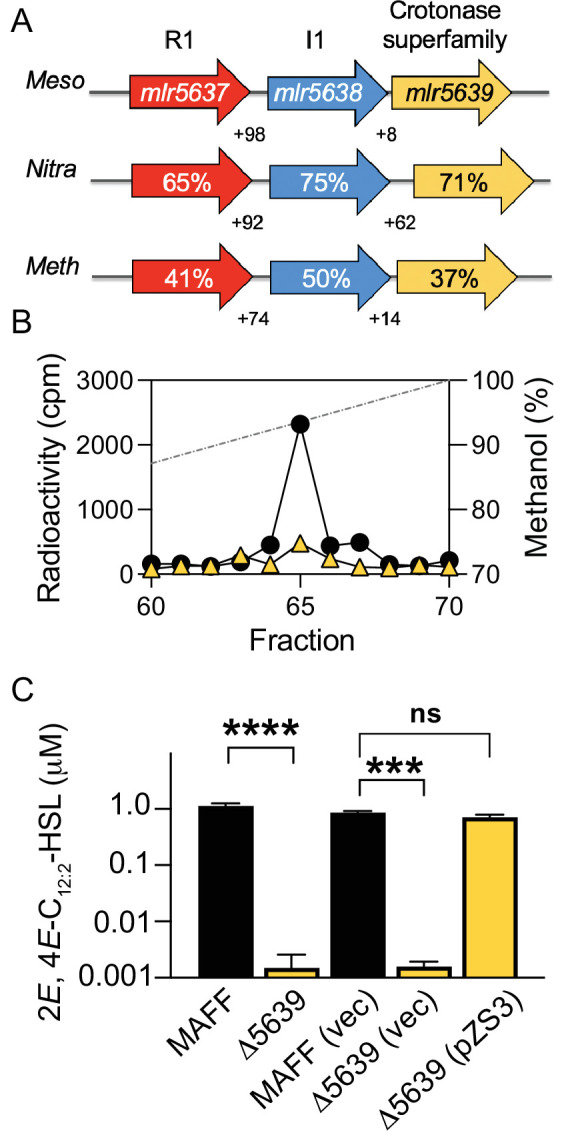
Influence of *mlr5639* on 2*E,* 4*E*-C_12:2_-HSL production. (**A**) The genomic regions of *R1-I1* in *M. japonicum* MAFF 303099 (*Meso,* top) and homologs in *Nitratireductor* strains StC3 and Z3-1 (*Nitra*, middle) and *Methylopila* sp. M107 (*Meth*, bottom). The MAFF 303099 locus designations are within the gene arrow (top), and the percentages in the homologous genes indicate the percent amino acid identity with the MAFF 303099 products. The positive numbers below the genes indicate the number of bases in the intergenic regions. (**B**) HPLC profile of ^14^C-AHLs in ethyl acetate extracts of culture fluid from *M. japonicum* MAFF 303099 wild type (black circles) or *mlr5639* mutant (yellow triangles). The dashed line shows the methanol gradient. ^14^C-labeling was performed twice for each strain with similar results, a representative experiment is shown. (**C**) 2*E,* 4*E*-C_12:2_-HSL levels in fluid from early stationary phase cultures of MAFF 303099 wild type (black, MAFF) or *mlr5639* mutant (yellow, Δ5639) as measured by using the R1-specific bioassay. Where indicated, the following plasmids were present: pBBP_gdh_ (45) the vector control plasmid (vec) or the *mlr5639* complementation plasmid (pZS3). The data shown are the means of three biological replicates, and the error bars are the standard deviation. Student’s t-test differences between indicated samples are significant *P* < 0.0001 (****), significant *P* < 0.001 (***), or not significant (ns).

We believe that the I1 AHL synthase uses *S*-adenosylmethionine and 2*E,* 4*E*-C_12:2_-CoA as reaction substrates. It is not clear as to how the CoA substrate might result from normal cellular metabolism. Our attention was drawn to the gene adjacent to *I1*. This gene, *mlr5639*, is separated from *I1* by eight base pairs and oriented in the same direction as *I1* (Fig. 5A). It seems likely that the two genes are co-transcribed. This gene encodes a polypeptide annotated as a member of the crotonase superfamily (Fig. 5A). Members of the crotonase superfamily are enzymes that catalyze diverse reactions but share a requirement to stabilize enolate anion intermediates derived from acyl-CoA substrates [reviewed in (44)]. Based on current knowledge about AHL synthases, might the downstream gene be involved together with *I1* in 2*E,* 4*E*-C_12:2_-HSL production? To address this question, we constructed a *mlr5639* deletion mutant and found that it produced little 2*E,* 4*E*-C_12:2_-HSL as measured by either ^14^C-radiolabeling (Fig. 5B) or the *P. putida* (pZS1) bioassay (Fig. 5C). The *mlr5639* mutation was complemented by providing an intact copy of *mlr5639* on a plasmid (pZS3, Fig. 5C). We note that even though we used the *I1* promoter to drive *mlr5639* expression, cells containing this plasmid grew slowly compared with vector control cells.

### The *R1-I1-*crotonase superfamily gene three-gene element occurs in some other genera of *Alphaproteobacteria*

The R1-I1 circuit is quite conserved in the genus *Mesorhizobium*, as homologs with high identity to R1-I1 occur in approximately 90% of the nearly 500 *Mesorhizobium* genome sequences deposited in the Joint Genome Institute Integrated Microbial Genomes (JGI IMG) repository (46). By using the JGI IMG “top homolog” search feature, we identified I1 homologs (50% amino acid identity or higher) in two genera in addition to *Mesorhizobium*. We found genes homologous to the *I1*-linked crotonase superfamily gene adjacent to *I1* (Fig. 5A). Thus, it seems as though this three-gene AHL QS circuit is not restricted to the genus *Mesorhizobium*. It is of some interest to determine whether these additional bacteria produce an AHL with *trans* unsaturated double bonds, perhaps 2*E,* 4*E*-C_12:2_-HSL itself.

## DISCUSSION

We present several lines of evidence that *M. japonicum* MAFF 303099 produces 2*E,* 4*E*-C_12:2_-HSL as the major AHL in our laboratory cultures. If other AHLs are produced, they are in very small amounts. Of the four R-I circuits in this strain, I1 is responsible for *2E, 4E*-C_12:2_-HSL production, and R1 is the transcription factor that responds to 2*E,* 4*E*-C_12:2_-HSL. Apparently, the other QS circuits are silent.

The discovery of 2*E,* 4*E*-C_12:2_-HSL adds to the list of *Alphaproteobacteria* shown to produce unsaturated AHLs as a major signal. This list includes *Rhizobium leguminosarum* (3-hydroxy-7*Z-*C_14:1_-HSL [47, 48]), *Rhodobacter sphaeroides* (7*Z*-C_14:1_-HSL [49]), *Methylorubrum extorquens* (*2E,* 7*Z*-C_14:2_-HSL [50, 51]), a marine *Mesorhizobium* (3-oxo-5*Z-*C_12:1_-HSL and 5*Z-*C_12:1_-HSL [13]), *Dinoroseobacter shibae* (2*E,* 11*Z*-C_18:2_-HSL [52]) as well as a *Phaeobacter* and several *Roseobacter* strains that produce 2*E,* 5*Z*-C_12:2_-HSL (38, 53). Interestingly, 3-oxo-C_12:2_-HSL has been found as the major AHL in human fecal samples (54). The positions of the unsaturated carbons and their isomeric arrangement in the fecal AHL were not determined. Levels of the 3-oxo-C_12:2_-HSLs in fecal samples from patients with flares of inflammatory bowel disease (IBD) are lower than in healthy patients or IBD patients in remission. The bacteria responsible for 3-oxo-C_12:2_-HSL production have not yet been identified (54). With our discovery that a plant-associated bacterium produces 2*E,* 4*E*-C_12:2_-HSL, it is of some interest to determine the isomeric arrangement of the two double bonds in the acyl side chain of the fecal AHL and to identify the bacteria responsible for its production. As discussed above, long-chain AHLs with unsaturated carbons in the acyl tail are common in *Alphaproteobacteria*. This may provide a clue as to the identity of the species responsible for the 3-oxo-C_12:2_-HSL found in fecal samples.

For most AHLs, high-resolution LC-MS/MS shows characteristic homoserine lactone fragments. These fragments were not evident for 2*E,* 4*E*-C_12:2_-HSL (Fig. 2B; Table S2) with the quadrupole time-of-flight (QTOF) MS system we employed. This is also true of some other AHLs with an unsaturation between carbons 2 and 3 (25, 26, 50), whereas other AHLs with the same unsaturations do show the characteristic 102 fragment (38, 51). We note this because LC-MS/MS often relies on identification of characteristic AHL fragments for discovery (11, 28, 29). Obviously, if we relied on this approach, we would not have found 2*E,* 4*E*-C_12:2_-HSL in *M. japonicum* MAFF 303099 cultures. As an example of the difficulty in relying on MS2 fragmentation approaches, Yang et al. (11) did not detect 2*E,* 4*E*-C_12:2_-HSL in *M. loti* NZP 2213 culture extracts, whereas we did (Fig. 4). Instead they identified C_12_-HSL as the NZP 2213 I1 product (11) likely due to using an AHL reporter sensitive to trace amounts of C_12_-HSL (14) and a reliance on an MS2 102 fragment in MS experiments. We note that Yang et al. (11) found that a *M. loti* NZP 2213 *I1* mutant formed fewer nodules on the roots of *L. japonicus* than the wild type. Thus, our findings implicate 2*E,* 4*E*-C_12:2_-HSL in root nodulation.

We showed that the *M. japonicum* MAFF 303099 R1 has a robust response to 2*E,* 4*E*-C_12:2_-HSL. It responds to 2*E,* 4*E*-C_12:2_-HSL concentrations as low as 1 nM (Fig. 3A), but it shows little or no response to the panel of other AHLs we tested. LuxR homologs have varying degrees of specificity. Some respond to a wide variety of AHLs, and others have a more restricted selectivity (43). The R1 response is at the extreme end of the LuxR homolog selectivity spectrum. This selectivity could provide protection against inappropriate responses to AHLs produced by other bacterial species in the rhizosphere, whereas bacteria with more promiscuous LuxR homologs might engage in AHL cross talk (43). It is important to mention that the extreme selectivity of the R1-I1 circuit could be useful in a variety of synthetic biology applications where cross talk can be an obstacle (55).

The unusual nature of the fatty acyl moiety of 2*E,* 4*E*-C_12:2_-HSL raised a question as to how might the CoA substrate for the I1 enzyme be produced. Several other CoA-dependent AHL synthases rely on normal cellular metabolites and CoA ligases to manufacture substrates such as phenylacetyl-CoA or isovaleryl-CoA (24). One interesting case is that of *R. palustris*, which relies on the exogenous addition of *p*-coumaric acid and cellular *p-*coumaroyl-CoA ligase activity to produce *p*-coumaroyl-HSL (25). Here we report a new twist on the theme. There is a gene adjacent to *I1* that we show is critical to I1-dependent 2*E,* 4*E*-C_12:2_-HSL synthesis. The product of this gene has been annotated as a member of the crotonase enzyme superfamily. We hypothesize that the gene product is needed for the synthesis of the CoA substrate for the *I1* gene product. We further hypothesize that the substrate is 2*E,* 4*E*-C_12:2_-CoA. Further biochemical studies are required to establish the role of the downstream gene in AHL production. We note that whole-genome sequencing has revealed similar gene arrangements in a few other bacterial species. This also merits further investigation. Regardless, we have shown that the basic QS circuit in *M. japonicum* MAFF 303099 involves three genes dedicated to the production of 2*E,* 4*E*-C_12:2_-HSL. This is a variation of the canonical two-gene R-I circuits. We consider this to be a circuit involving three linked genes.

We are left with many questions about R1-I1-“crotonase” type QS in the genus *Mesorhizobium*. In what way does the *I1*-linked crotonase superfamily gene contribute to 2*E,* 4*E*-C_12:2_-HSL production? What genes are controlled by this AHL circuit, and in what way do they influence nodulation of host plants? Under what conditions might the other three AHL QS systems of *M. japonicum* MAFF 303099 be active? Ramsay and colleagues have shown that in *M. japonicum* R7A, the *R3-I3* homologs are repressed by an epigenetically controlled anti-activator (56, 57), does a similar regulatory control exist in MAFF 303099? What is the relationship of this system to the unknown system that results in 3-oxo-C_12:2_-HSL accumulation in human fecal samples?

## MATERIALS AND METHODS

### Bacterial strains, growth conditions, and chemicals

We used the following bacterial species and strains: *M. japonicum* MAFF 303099 (19); the MAFF 303099 *I1* (*mlr5638*) transposon mutant 27T02f03 (22); *Mesorhizobium* AP09 (36); *M. loti* NZP 2213 (11); *Pseudomonas putida* F1 (58); and *E. coli* NEB 5α (New England Biolabs, Ipswich, MA) and S17-1 (59). *Mesorhizobium* strains were grown at 30°C with shaking in tryptone yeast extract broth (TY) (60) or, where indicated, modified Vincent’s minimal medium (BVM) (61, 62) where arabinose was replaced with 0.5% glucose or ^13^C-U-glucose (Cambridge Isotope Laboratories, Andover MA). *M. loti* NZP 2213 cultures required the addition of 50 mM 3-morpholinopropane-1-sulfonic acid to maintain a neutral pH. *E. coli* and *P. putida* were grown in lysogeny broth (LB) (63) at 37°C and 30°C, respectively. When required, the following antibiotics were used: gentamicin (10 µg/mL *E. coli*, 30 µg/mL *P*. *putida,* and *M. japonicum*), ampicillin (100 µg/mL *E. coli*), and fosfomycin (50 µg/mL *Mesorhizobium* strains). For plating, media contained 1.5% agar. The following synthetic AHLs were purchased commercially (Cayman Chemical, Ann Arbor, MI, USA): C_12_-HSL, 3-oxo-C_12_-HSL, 3-hydroxy-C_12_-HSL, C_10_-HSL, C_14_-HSL, 3-hydroxy-7*Z*-C_14:1_-HSL, 3-oxo-7*Z*-C_14:1_-HSL, and 9*Z*-C_14:1_-HSL. Chemically synthesized 2*E*, 4*E*-C_12:2_-HSL was generously provided by Professor Stefan Schultz (Technische Universität Braunschweig).

### Identification and classification of *Mesorhizobium* quorum sensing genes

We identified QS genes in genomes hosted on the JGI IMG website (46) by searching for pfam motifs specific to LuxI (pfam00765) and LuxR (pfam03472 and pfam00196) homologs (64). The JGI IMG database resource also identifies some LuxI homologs as CoA-utilizing enzymes based on the integrated Kyoto Encyclopedia of Genes and Genomes orthology and Enzyme Nomenclature (EC) database (65 - 67) categories (K18096 and EC 2.3.1.228/229, respectively) as we have done previously (24).

### Radiotracer analysis

We used a ^14^C-radiotracer assay (27, 30) to detect AHLs produced by *Mesorhizobium* strains. For experiments shown in Fig. 1A, we modified a previous protocol (68) as follows: cells were grown in 5 mL of TY broth to mid-logarithmic phase (OD_600_ of 0.7–0.9), harvested by centrifugation, and resuspended in 2 mL of phosphate-buffered saline (69) containing 0.5% (wt/vol) glucose and 5 µCi of L-[1-^14^C]-methionine (55 mCi per mmol; American Radiolabeled Chemicals, Inc., St. Louis, MO, USA). Cultures were split, and AiiA lactonase (100 µg/mL final concentration) was added to one of the 1 mL samples (MalE-AiiA enzyme was a gift from *N*. Smalley, prepared as described in [70]). After further incubation at 30°C for 4–7 h, AHLs were extracted from the cell suspensions with two equal volumes of acidified ethyl acetate (0.1 mL glacial acetic acid per liter), and the extracts were then fractionated by C_18_ reverse-phase HPLC in a 10%–100% methanol gradient (0.75 mL/min). One-minute fractions were collected, mixed with 4 mL of 3a70b scintillation fluid (Research Products International, Mount Prospect IL), and radioactivity was determined by liquid scintillation counting. For experiments shown in Fig. 5B, we modified a previous protocol (7), as follows: *M. japonicum* MAFF 303099 and the *mlr5639* crotonase mutant were grown in BVM glucose medium to mid-logarithmic phase (OD_600_ of 0.65–0.75). Then 5 µCi of L-[1-^14^C]-methionine and 2 µM chemically synthesized 2*E*, 4*E*-C_12:2_-HSL (to ensure activation of *I1* gene transcription) were added to 5 mL cultures. After 16–18 h at 30°C with shaking culture fluid was extracted with acidified ethyl acetate. Ethyl acetate extracts were fractionated by HPLC and radioactivity was quantitated as described above.

### Plasmid and strain construction

We used *E. coli* DH5α-mediated assembly of PCR fragments (71) to create pZS1, by analogy to the phenylacetyl-HSL reporter plasmid pLL1 (24). The pZS1 construct contains the R1 gene (*mlr5637*) driven by a *gdh* constitutive promoter (45) and a *I1-mCherry* transcriptional fusion. The pZS2 plasmid is pZS1 with 580 bp of the 5′ end of *R1* (79% of the 732 bp gene) removed by using a similar protocol. Because there is only 8 bp in the intergenic region between *I1* and *mlr5639* (Fig. 5A), we presume they are co-transcribed from the *I1* promoter. Therefore, we created the crotonase superfamily gene complementation plasmid (pZS3) containing the *mlr5639* open reading frame driven by the *I1* (*mlr5638*) promoter and an *I1_13-580_* deletion. Plasmids were introduced into bacteria (*E. coli* or *P. putida*) by electrotransformation, and transformants were selected by plating on gentamicin-containing agar. Plasmid sequences were confirmed by whole-plasmid sequencing by a commercial vendor (Plasmidsaurus, Eugene OR), and the sequences deposited at National Center for Biotechnology Information (Genbank OQ303973, OQ303974, and OQ822817). The *mlr5639* knockout construct was created by synthesizing a gBlock (Integrated DNA Technologies, Coralville, IA, USA) comprising 686 bp upstream and 666 bp downstream flanking DNA sequence, which created an in-frame deletion leaving the first 16 and last 5 codons of the *mlr5639* open reading frame. This fragment was cloned into the suicide vector pJQ200SK (72), transferred to *E. coli* S17-1 by transformation, and then mobilized into MAFF 303099 by conjugation. Recombinants were selected by plating on gentamicin and fosfomycin, and double crossovers were selected by growth on 10% sucrose and loss of gentamicin resistance.

### AHL bioassays

Detection of 2*E*, 4*E*-C_12:2_-HSL from ethyl acetate extracts of culture fluid or HPLC fractions of extracts was by using *P. putida* (pZS1) as follows: cell culture fluid extracts, HPLC fractions, or chemically synthesized AHLs were added to 13-mm glass tubes, and the solvent removed by evaporation under a stream of N_2_ gas. Then 0.3 mL of *P. putida* (pZS1), diluted 1:100 from an overnight culture into fresh LB broth plus gentamicin, was added to each tube. After 16 h at 30°C with shaking, we transferred 100 or 150 µL samples to wells of 96-well black microtiter dish plates, and mCherry fluorescence (587 nm excitation, 610 nm emission, and gain 100) was measured by using a Synergy H1 plate reader (Biotek Instruments, Winooski, VT, USA). Standard curves were generated by measuring the fluorescence response to synthetic 2*E*, 4*E*-C_12:2_-HSL (linear response was between 1 and 10 nM). To detect any C_12_-HSL in NZP 2213 extracts, we utilized the QscR-based reporter strain *E. coli* (pJNQ pPROBE-P_PA1897_) as described previously (43), except that cells were incubated in 13-mm glass tubes rather than 96-deep well plates, and we used a fluorescence gain setting of 75. Note that *E. coli* (pJNQ pPROBE-P_PA1897_) can detect 2*E*, 4*E*-C_12:2_-HSL but only at micromolar concentrations.

### Purification and identification of C_12:2_-HSL

We purified AHLs extracted from culture fluid after the removal of cells by centrifugation. Purification was guided by using the *P. putida* (pZS1) bioassay. Extraction, preparation, and HPLC fractionation were similar to that described elsewhere (7). We used the 75% and 100% methanol cuts from the C_18_ Sep-pak cartridge (Waters, Milford, MA, USA). For HPLC, the flow rate was 0.75 mL/min, and the isocratic separation step was in 67% methanol. LC-MS/MS was performed on an AB Sciex 5600 QTOF (AB Sciex, Framingham, MA, USA) at the Department of Medicinal Chemistry, University of Washington. Samples were run as delivered by LC gradient over an Aquity UPLC HSS T3 column (100 Å, 2.1 × 100 mm; Waters, Milford, MA, USA), with a gradient from 5% methanol in water to 100% methanol (with 0.1% formic acid) over 13 min with a 0.2 mL/min flow rate. NMR analysis was performed at the University of Utah on a Direct Drive 500 MHz instrument (Agilent Technologies, Santa Clara, CA, USA) with a high sensitivity cold probe detection system. Spectroscopy scans were generated by using a DU 800 spectrophotometer (Beckman Coulter, Brea, CA, USA).

## SUPPLEMENTAL MATERIAL

Supplemental File mbio.01010-23-s0001.docx All supplemental files—Index, three tables, five figures, and literature cited.

ASM does not own the copyrights to Supplemental Material that may be linked to, or accessed through, an article. The authors have granted ASM a non-exclusive, world-wide license to publish the Supplemental Material files. Please contact the corresponding author directly for reuse.
