# Supplementary material for: A Mesorhizobium japonicum quorum sensing circuit that involves three linked genes and an unusual acyl-homoserine lactone signal
Source: mBio. 2023 May 25;14(4):e01010-23. doi: 10.1128/mbio.01010-23 (PMC10470506; doi:10.1128/mbio.01010-23)
Supplement: Supplemental File — All supplemental files—Index, three tables, five figures, and literature cited. [file mbio.01010-23-s0001.docx]

*Supplemental index for:*

**A *Mesorhizobium japonicum* quorum sensing circuit that involves three linked genes and an unusual acyl-homoserine lactone signal**

Zehui Suo, Dale A. Cummings Jr., Aaron W. Puri, Amy L. Schaefer, and E. Peter Greenberg

**Table S1**. Information on quorum sensing genes, products, and phenotypes in *Mesorhizobium* page 2

**Table S2.** MS2 peak list for chemically synthesized vs. purified 2*E,* 4*E-*C_12:2_-HSL page 3

**Table S3.** NMR assignments for purified 2*E*, 4*E*-C_12:2_-HSL page 4

**Figure S1.** ^1^H NMR spectrum of purified 2*E*, 4*E*-C_12:2_-HSL page 5

**Figure S2.** ^13^C NMR spectrum of purified 2*E*, 4*E*-C_12:2_-HSL page 6

**Figure S3.** COSY NMR spectrum of purified 2*E*, 4*E*-C_12:2_-HSL page 7

**Figure S4.** HSAQCAD NMR spectrum of purified 2*E*, 4*E*-C_12:2_-HSL page 8

**Figure S5.** HMBCAD NMR spectrum of purified 2*E*, 4*E*-C_12:2_-HSL page 9

**Appendix Literature Cited** page 10

**Table S1**. Information on quorum sensing genes, products, and phenotypes in the genus *Mesorhizobium*

| Species or strain^a^ | *luxI* homolog and  gene/locus (% ident)^b^ | paired *luxR* homolog and gene/locus (% ident)^b^ | | Reported AHL | Associated mutant phenotype | Reference |
| --- | --- | --- | --- | --- | --- | --- |
| *Mesorhizobium japonicum*  MAFF 303099 | *I1* *mlr5638*  *I2* *mlr6103*  *I3* *mlr6385*  *I4* *mlr9546* | *R1 mlr5637*  *----*  *R3 mlr6102*^c^  *R4 mlr9544­­­­* | | 2*E,* 4*E*-C_12:2_-HSL^d^  ND^ef^  ND^f^  ND^f^ |  | This work  This work  This work  This work |
| *Mesorhizobium loti*  NPZ 2213 | *mlrI1* (95% I1)  *mlrI3* (92% I2)  *mlrI2* (100% I3) |  | | C_12_-HSL^g^  2*E,* 4*E*-C_12:2_-HSL^h^  ND^g^  3oxoC_6_-, C8-,  C10-HSL^g^ | Reduced nodulation efficiency  Reduced nodulation efficiency  Reduced nodulation efficiency | (1)  This work  (1)  (1) |
| *Mesorhizobium japonicum*  R7A | *R7A2020_RS31320* (100% I1)  *traI2* (90% I2)  *traI1* (99% I3) | *R7A202_RS31325* (99% R1)  *----*  *traR* (98% R3) | | Not tested  ND^i^  3oxoC_6_-HSL^i^ | Reduced integrative and conjugative element transfer | (2)  (2) |
| *Mesorhizobium* sp. ORS 3359 | *MPLA_1690004* (82% I1)  *----*  *mesI* (90% I4)  *MPLA_2140008* | | *MPLA_1690004* (85% R1)  *MPLA_750127* (92% R3)  *MPLA_2130040* (78% R4)  *MPLA_2140009* | Not tested  Not tested  C_6_-HSL^j^  Not tested |  | (3) |
| *Mesorhizobium* sp. AP09 | *Ga0215757_5903* (99% I1) | *Ga0215757_5902* (97% R1) | | 2*E,* 4*E*-C_12:2_-HSL^f^ |  | This work |

^a^NCBI Genbank ID for those strains with available sequenced genomes: BA000012 (MAFF 303099), CP051772 (R7A), CCNC00000000 (ORS 3359), and QRAR00000000.1 (AP09)

^b^Name of published *luxI/luxR* homolog or locus number (% amino acid identity with the corresponding MAFF 303099 homolog is indicated in parentheses)

^c^Paired *luxR* homolog assignment based on work with *M. japonicum* R7A (2)

^d^As assessed by AHL radiolabel assay, R1-specific bioassay, mass spectrometry and NMR

^e^Not detected

^f^As assessed by AHL radiolabel assay

^g^As assessed by mass spectrometry, heterologous expression in *E. coli*, and relaxed-specificity bioassay

^h^As assessed by R1-specific bioassay and HPLC elution

^i^As assessed by mass spectrometry and relaxed-specificity bioassay

^j^As assessed using *in vitro* experiments with purified enzyme and chemically synthesized CoA substrates

**Table S2.** High resolution MS2 peak list for chemically synthesized vs. purified 2*E,* 4*E-*C_12:2_-HSL (M+H). The 20 highest intensity signals are shown. Both the synthesized and purified parent compounds eluted at 15.88 minutes on the liquid chromatography gradient as described in the MATERIALS AND METHODS.

| Synthesized | | Purified | |
| --- | --- | --- | --- |
| *­­­m/z* | intensity | *m/z* | intensity |
| 81.03401 | 16739 | 81.03401 | 14413 |
| 179.14566 | 10055 | 179.14377 | 9227 |
| 53.03847 | 9604 | 53.03847 | 8814 |
| 95.04909 | 6305 | 95.04909 | 5603 |
| 55.0551 | 5693 | 55.05405 | 4797 |
| 79.05519 | 4520 | 79.05393 | 3794 |
| 67.05558 | 4025 | 67.05442 | 3339 |
| 97.10346 | 3229 | 97.10067 | 2837 |
| 161.13386 | 2704 | 161.13386 | 2505 |
| 83.05017 | 1907 | 119.08482 | 1660 |
| 119.08637 | 1869 | 83.05017 | 1582 |
| 91.05481 | 1704 | 107.08521 | 1515 |
| 107.08667 | 1664 | 280.19402 | 1490 |
| 105.07066 | 1472 | 105.06921 | 1332 |
| 93.06986 | 1429 | 91.05346 | 1223 |
| 280.19402 | 1377 | 69.07033 | 1196 |
| 69.07033 | 1216 | 95.08492 | 1172 |
| 95.08492 | 1076 | 93.06986 | 1154 |
| 73.06602 | 1004 | 73.06482 | 998 |
| 81.06963 | 925 | 133.09899 | 970 |

**Table S3.** NMR assignments for purified 2*E*, 4*E*-C_12:2_-HSL in CDCl_3_ (500 MHz)

| **Position** | **𝜹_c_, type** | **𝜹_H_ (*J* in Hz)** |
| --- | --- | --- |
| 4’ | 33.6, CH_2_ | 2.20, 2.94, m |
| 3’ | 51.80, CH | 4.63, dd (5.4, 1.4) |
| 2’ | 178.14, C |  |
| 5’ | 68.87, CH_2_ | 4.50, 4.33, m |
| 1 | 169.46, C |  |
| 2 | 122.58, CH | 5.80, d (15) |
| 3 | 145.71, CH | 7.24, dd, (15, 10.5) |
| 4 | 130.66, CH | 5.95, dd, (15.5, 10) |
| 5 | 147.49, CH | 6.17 dt, (15.5, 11) |
| 6 | 35.68, CH_2_ | 2.19, td, (5.5, 5) |
| 7 | 31.80, CH_2_ | 1.45, m |
| 8 | 31.76, CH_2_ | 1.42, m |
| 9 | 31.38, CH_2_ | 1.33, m |
| 10 | 34.44, CH_2_ | 1.29, m |
| 11 | 25.30, CH_2_ | 1.27, m |
| 12 | 16.76, CH_3_ | 0.90, t (7.5) |
| NH (7’) |  | 5.95 |

**Figure S1.** ^1^H NMR spectrum of purified 2*E*, 4*E*-C_12:2_-HSL in CDCl_3_ (500 MHz)

**Figure S2.** ^13^C NMR spectrum of purified 2*E*, 4*E*-C_12:2_-HSL in CDCl_3_ (500 MHz)

**Figure S3.** COSY NMR spectrum of purified 2*E*, 4*E*-C_12:2_-HSL in CDCl_3_ (500 MHz)

**Figure S4.** HSAQCAD NMR spectrum of purified 2*E*, 4*E*-C_12:2_-HSL in CDCl_3_ (500 MHz)

**Figure S5.** HMBCAD NMR spectrum of purified 2*E*, 4*E*-C_12:2_-HSL in CDCl_3_ (500 MHz)

**Appendix Literature Cited**

1. Yang M, Sun K, Zhou L, Yang R, Zhong Z, Zhu J. 2009. Functional analysis of three AHL autoinducer synthase genes in *Mesorhizobium loti* reveals the important role of quorum sensing in symbiotic nodulation. Can J Microbiol 55:210-4.

2. Ramsay JP, Sullivan JT, Jambari N, Ortori CA, Heeb S, Williams P, Barrett DA, Lamont IL, Ronson CW. 2009. A LuxRI-family regulatory system controls excision and transfer of the *Mesorhizobium loti* strain R7A symbiosis island by activating expression of two conserved hypothetical genes. Mol Microbiol 73:1141-55.

3. Dong SH, Nhu-Lam M, Nagarajan R, Nair SK. 2020. Structure-guided biochemical analysis of quorum signal synthase specificities. ACS Chem Biol 15:1497-1504.
